# The effect of curcumin supplementation on anthropometric indices, insulin resistance and oxidative stress in patients with type 2 diabetes: a randomized, double-blind clinical trial

**DOI:** 10.1186/s13098-019-0437-7

**Published:** 2019-05-27

**Authors:** Homa Hodaei, Mahsa Adibian, Omid Nikpayam, Mehdi Hedayati, Golbon Sohrab

**Affiliations:** 1 Clinical Nutrition and Dietetics Department, Faculty of Nutrition Sciences and Food Technology, National Nutrition and Food Technology Research Institute, Shahid Beheshti University of Medical Sciences, 9, Hafezi St., Farahzadi Blvd., Shahrak Qods, P.O. Box: 19395-4741, Tehran, Iran; 2 Talented Student Center, Student Research Committee, Nutrition Research Center, Tabriz University of Medical Sciences, Tabriz, Iran; 3 Cellular & Molecular Research Center, Research Institute of Endocrine Sciences, Shahid Beheshti University of Medical Sciences, Tehran, Iran

**Keywords:** Curcumin, Anthropometric, Insulin resistance, Type 2 diabetes

## Abstract

**Background:**

Diabetes mellitus is a common metabolic disorders in human and affect a lot of people around the world. Curcumin is a component of turmeric and in many studies therapeutic effects such as anti-hypertensive, anti-hyperlipidemia, anti-hyperglycemia for this substance are shown.

**Aim:**

The aim of this study was to investigate the effect of curcumin supplementation on anthropometric indices glycemic control and oxidative stress in overweight patients with type 2 diabetes.

**Materials and methods:**

In this randomized, double-blind, placebo-controlled trial, 53 participants with type 2 diabetes were divided randomly into the experimental and control groups to receive either 1500 mg curcumin or placebo capsule three times in a day for 10 weeks.

**Result:**

Supplementation with curcumin in type 2 diabetes compare to placebo causes a significant changes in mean weight (− 0.64 ± 0.22 vs. 0.19 ± 0.37 p < 0.05), body mass index (BMI) (0.3 ± 0.03 vs. 0.1 ± 0 p < 0.05), waist circumference (WC) (− 1.2 ± 0.4 vs. − 0.43 ± 0.11 p < 0.05) and fasting blood sugar (FBS) (− 7 ± 2 vs. 3 ± 0.2 p < 0.05) but did not show any difference for hemoglobin A1c (HbA1c), insulin, malondialdehyde (MDA), total antioxidant capacity (TAC), Homeostatic Model Assessment for Insulin Resistance (HOMA-IR) and pancreatic B cell function (HOMA-B) at end of study.

**Conclusion:**

This study indicated that daily administration of 1500 mg curcumin has positive effects in reducing fasting blood glucose and weight in patients with type 2 diabetes.

*Trial registration* NCT02529982. Registered 19 August 2015, http://www.clinicaltrial.gov

## Background

Diabetes mellitus is the most common and important metabolic disorders in human. According to World Health Organization (WHO) report in 2016, 422 million people in world are diagnosed with diabetes, although the prevalence of this disorder is on the rise, especially in Asian countries [1]. Also recent researches showed that obesity affects more than 600 million people in worldwide. Obesity and overweight are two major risk factors for diabetes and its complications. Investigations show that almost 90% of diabetic patients are overweight. Obesity could affect the metabolism in the body by insulin resistance and reducing insulin secretion [2].

Curcumin is the most important component of turmeric (curcuma longa) and has a yellow color. Curcumin commonly used as a food additive in Asian countries [3]. Also this substance has a Therapeutic characteristics and had role in traditional medicine as medicinal herb in india and china [4]. Curcumin can cause a significant reduction on total cholesterol, blood pressure, aggregation platelet, myocardial infraction and prevention from thrombosis, rheumatoid arthritis [5, 6].

In some clinical trials Desirable effects of curcumin has shown to be a therapeutic agent for treatment of diabetes and complications of High blood glucose. Curcumin as an antioxidant and anti-inflammatory agent has different effects on diabetes through reducing death of beta cells, improving its function and prevent insulin resistance in rodent models [7–11]. Also curcumin thanks to anti-inflammatory effect has positive impression on weight loss. Give that obesity is associated with chronic inflammation, curcumin as an anti-inflammatory phytochemical has been studies [12].

Most of studies conducted for investigated effects of curcumin is on animal model [9, 13–20] and studies in human model is rare and many effects of this substance in humans is undiscovered. Therefore the aim of this study was to investigate the effects of curcumin supplementation on glycemic control, oxidative stress and anthropometric indices in patients with type 2 diabetes.

## Method and materials

### Participants

All of the participants in this study were adult patients who had come to the health centers of district 2 of Tehran/Iran. Patients with noninsulin dependent diabetic, aged between 40 and 70 years old and BMI 18.5–35 kg/m^2^ with a diagnose of 1 to 10 years participant in this study. Patients were excluded from the trail if they were suffering from inflammatory, hepatic and renal disease, and receiving multivitamin and mineral supplements or herbal medicines during last 3 months. They were informed about aims of study and written informed consent was obtained from all of them. Patients were educated not to change their usual dietary habits, physical activity, lifestyle and drugs during the intervention period otherwise, they were excluded from the study. This randomized, double-blinded, placebo-controlled trial was conducted at National Nutrition And Food Technology Research Institute of Shahid Beheshti University. This clinical trial has been registered in the clinical Trail.gov with the following identification: NCT02529982.

### Study design

This study was a randomized, double blind, placebo-controlled trial. Patients were matched for gender and then randomly allocated to the curcumin group (n = 25) meals or placebo group (n = 28) respectively for consume three capsules, of 500 mg of curcumin and placebo after each main mail in three doses by block randomization for 10 weeks. Curcumin and placebo capsules were prepared by Arjuna National Extract Ltd company of India. Based on studies receiving 7 gr curcumin or placebo do not increase blood glucose in patients with diabetes [12]. Each curcumin capsules had curcuminoid content of 440 mg (347 mg curcumin, 84 mg desmethoxycurcumin and 9 mg bis-desmethoxycurcumin) and 38 mg of turmeric oil. Placebo capsule had the same shape and color of curcumin capsule and contain 444 mg of cooked rice flour. Random allocation of patients were performed by a third person. Throughout the course of the intervention researchers and participants were not aware about allocation.

### Follow up

In order to control patients for taking supplements and placebo they were followed-up by phone every 15 days and Supplement and placebo were given to the patients for a period up to the middle of the study, and patients were asked to bring the bottle of capsules in their next visit for assessing their compliance and if anyone used less than %90 of capsules he would be excluded.

### Measurement of anthropometric parameters and blood pressure

Anthropometric measurements, including weight, height, waist and hip circumferences were measured at baseline and at the end of the study. The weight of each patient was measured without shoes and light clothing by using seca scale with the precision of 100 gr and height was also measured by using a stadiometer without shoes with precision of 0.5 cm. To measure waist circumference tape measure was placed about halfway between the bottom of the lowest rib and the top of hip bones, and to measure hip circumference tape measure was placed in widest part of hip, each measurements was repeated twice. Body mass index (BMI) was calculated as weight (kg) divided by square of height (m). Systolic blood pressure (SBP) and diastolic blood pressure (DBP) were measured twice in a sitting position on the right arm after 15 min of rest and the mean of the two measurements was considered as the subject’s blood pressure.

### Assessment of dietary and physical activity

Dietary intake of each participant in this study assessment at baseline, at the 5th week and end of study by 3 day records, patients were enlighten about portion size and how to record their dietary intake and then all records converted to grams and analyzed by using Nutritionist IV software (N Squared Computing, San Bruno, CA, USA). To assess physical activity during intervention, valid physical activity questionnaire was completed for all participates at the begging and the end of study [21].

### Measurement of biochemical parameters

A sample of 10 ml blood was taken from each patients after 12–14-h fasting at baseline and the end of study. Serum concentration of glucose, total antioxidant capacity (TAC), and Malondialdehyde (MDA) were determined by using colorimetric method (Zellbio, Germany). Insulin level was measured by ELISA method, and Hemoglobin A1c (HbA1c) level was measured by chromatography method (Zellbio, Germany). Levels of Change in pancreatic B-cell function (HOMA-B) and Homeostatic Model Assessment for Insulin Resistance (HOMA-IR) were calculated by formulas respectively, HOMA-B = 20 × Insulin(µU/ml)/glucose(mg/dl) − 3.5 and HOMA-IR = (glucose(mg/dl) × Insulin(µU/ml))/22.5 [21].

### Statistical analysis

Data were analyzed by using SPSS 22 software. For comparison of quality variables Chi square tests were used between the two groups. Normality of quantity variables were assessed by Kolmogorov–Smirnov test. independent t-tests and paired t-tests were used to compare parameters between and within the groups, respectively. The results were displayed as mean ± SD, and a P-value < 0.05 was considered significant. The minimum number of patients estimated for each group was 22 at a power (1−β) 80% and α = 0.05 for this parallel study, for detect a difference of 1.7 mg/dl in fasting blood sugar concentration with a standard deviation of 1.8 mg/dl [18].

## Result

**Fig. 1:**
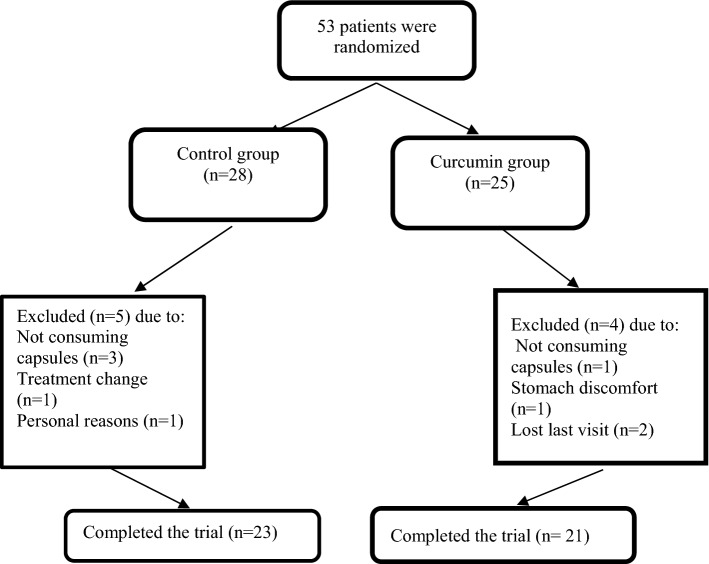
Flow chart of the study

Based on the flow chart of the trial 44 participates completed the study. 44 patients were randomly divided in curcumin group (n = 21) and placebo group (n = 23) (Fig. 1). None of the patients did not reported any serious side effect. There were no significant differences in baseline characteristics include sex distribution, smoking, mean age, duration of diabetes and drugs between the two groups at baseline and the end of week 10 (Table 1).

**Table 1:** Baseline characteristics of patients in curcumin group and placebo group

Variable	Curcumin group	Placebo group	P value
Sex (%)			
Male	61.6	39.1	0.2
Female	38.4	60.9	
Smoking (%)	19	13	0.4
Age (years)	58 ± 8	60 ± 7	0.09
Duration of diabetes (years)	8 ± 4.6	8 ± 7.9	0.7
Anti-hyperglycemic drugs metformin	52%	43%	0.8
Gelibenclamide	52%	43%	
Gelibenclamide + metformin	33%	35%	
Non	10%	17%	
Anti-hyperlipidemic drugs	47.6%	52.2%	0.6
Anti-hypertensive drugs	66.7%	65.2%	0.5

P values are for comparison of variable between curcumin and placebo group (all by Chi square analyzed except age and duration of diabetes which were analyzed by t-independent test, p: 0.05)

Statistical analysis could not show any significant differences between two groups with regard to physical activity, mean energy, the dietary intake of carbohydrate protein, fiber, total fat, SAFA, MUFA, PUFA, cholesterol, vitamin E and C and polyphenol intakes at baseline, week 5 and week 10 of the study (Tables 2 and 3).

**Table 2:** Dietary factors in curcumin group and placebo group

Factor	Groups	Basline	10 weeks	P-value	
				P*	P**
Calorie (Kcal/day)	Curcumin	1942 ± 485	1978 ± 458	NS	NS
	Placebo	1836 ± 683	1801 ± 578		
Protein (gr/day)	Curcumin	77 ± 24	82 ± 26	NS	NS
	Placebo	64 ± 18	73 ± 23		
Carbohydrate (gr/day)	Curcumin	257 ± 71	254 ± 71	NS	NS
	Placebo	247 ± 112	235 ± 88		
Fiber (gr/day)	Curcumin	28 ± 11	28 ± 12	NS	NS
	Placebo	26 ± 9	26 ± 11		
Total fat (gr/day)	Curcumin	74 ± 24	75 ± 21	NS	NS
	Placebo	71 ± 28	68 ± 21		
SAFA (gr/day)	Curcumin	20 ± 9	19 ± 5	NS	NS
	Placebo	19 ± 9	19 ± 11		
MUFA (g/d)	Curcumin	26 ± 9	28 ± 9	NS	NS
	Placebo	24 ± 7	25 ± 7		
PUFA (g/d)	Curcumin	17 ± 6	20 ± 7	NS	NS
	Placebo	17 ± 5	17 ± 4		
Cholesterol (mg/d)	Curcumin	260 ± 174	246 ± 174	NS	NS
	Placebo	205 ± 111	250 ± 123		
Vitamin E (mg/d)	Curcumin	22 ± 10	23 ± 9	NS	NS
	Placebo	20 ± 7	20 ± 5		
Vitamin C (mg/d)	Curcumin	135 ± 93	145 ± 105	NS	NS
	Placebo	113 ± 71	115 ± 71		

NS: Not significant

P*: p values are for comparison variables in one group (P: 0.05)

P**: p values are for comparison variables between tow group (P: 0.05)

**Table 3:** Polyphenol intakes in curcumin group and placebo group

Factor	Group	Basline	10 week	P value	
				P*	P**
Total polyphenol (mg/d)	Curcumin	1970 ± 485	1978 ± 458	NS	NS
	Placebo	1896 ± 483	1901 ± 445		
Flavonoids (mg/d)	Curcumin	1753 ± 420	1760 ± 420	NS	NS
	Placebo	1687 ± 389	1692 ± 415		
Phenolic acid (mg/d)	Curcumin	158 ± 46	158 ± 42	NS	NS
	Placebo	152 ± 51	152 ± 48		
Lignin, Stilbene and	Curcumin	59 ± 11	59 ± 13	NS	NS
other polyphenols (mg/d)	Placebo	57 ± 13	57 ± 11		

NS: Not significant

P*: p values are for comparison variables in one group (P: 0.05)

P**: p values are for comparison variables between tow group (P: 0.05)

At the end of the study mean body weight decreased significantly in curcumin group compared to the baseline (p = 0.01), and also analysis between two group had showed a significant reduce for mean body weight (p = 0.04). Also Mean BMI decreased significantly in curcumin group at the end of the study compared to the baseline (p = 0.03), however there was not any significant differences between two groups at the end of the stud. Mean hip circumference reduced significantly in curcumin group compared to baseline and placebo group (p = 0.05 and 0.01, respectively), but The mean waist circumference, systolic and diastolic blood pressure had no significant different between and within two groups (Table 4).

**Table 4:** Anthropometric measures and blood pressure in curcumin and placebo groups

Parameters	Time	Curcumin group (sd ± mean)	Control group (sd ± mean)	P2
Weight (kg)	Baseline	78 ± 13.28	74.04 ± 11.5	0.32
	Week 10	77 ± 13.6	74.23 ± 12.3	0.44
	P1	0.01	0.5	
	Mean changes	− 0.64 ± 0.22	0.19 ± 0.37	0.04
BMI (kg/m^2^)	Baseline	29.2 ± 3.76	28.2 ± 2.5	0.33
	Week 10	28.9 ± 3.73	28.1 ± 2.5	0.5
	P1	0.03	0.6	
	Mean changes	0.3 ± 0.03	0.1 ± 0	0.08
Hip circumference (cm)	Baseline	109 ± 8.34	107 ± 5.07	0.35
	Week 10	108 ± 8.02	107 ± 5.14	0.80
	P1	0.05	0.2	
	Mean changes	− 1 ± 0.32	0 ± 0.46	0.01
Waist circumference (cm)	Baseline	101 ± 8.52	97 ± 8.20	0.10
	Week 10	100 ± 8.92	96 ± 8.09	0.19
	P1	0.06	0.2	
	Mean changes	− 1.2 ± 0.4	− 0.43 ± 0.11	0.26
Systolic blood pressure (mmgh)	Baseline	12 ± 2.01	12 ± 1.49	0.86
	Week 10	12 ± 1.65	12 ± 1.66	0.52
	P1	0.91	0.37	
	Mean changes	0 ± 1.67	0 ± 1.41	0.62
Diastolic blood pressure (mmgh)	Baseline	8 ± 1.41	7.5 ± 1.12	0.53
	Week 10			
	P1	8 ± 1.25	7 ± 0.76	0.02
	Mean changes	0 ± 1.08	− 0.5 ± 1.02	0.13

P1: obtained from paired t-test (p: 0.05)

P2: obtained from Independent sample t-test (p: 0.05)

After 10 weeks intervention with curcumin mean serum concentration of fasting blood glucose decreased significantly in treatment group compared to placebo group at the end of the study (p = 0.02) but The mean serum concentrations of insulin, HbA1c, HOMA-IR, HOMA-B had no significant changes within each group or between groups during study and mean serum concentrations of MDA and TAC had no significant changes between two groups at the end of the study (Table 5).

**Table 5:** Serum concentrations of FBS, Insulin, HbA1c, MDA, TAC and measurements of HOMA-IR, HOMA-β in the curcumin treated and control group

Parameters	Time	Curcumin group (sd ± mean)	Control group (sd ± mean)	P2
FBS (mg/dl)	Baseline	160 ± 35	144 ± 40.6	0.28
	Week 10	153 ± 33	147 ± 40.4	0.67
	P1	0.05	0.3	
	Mean changes	− 7 ± 2	3 ± 0.2	0.02
Insulin (mU/L)	Baseline	9.2 ± 9	8.3 ± 6	0.70
	Week 10	9.4 ± 6	9.7 ± 4.7	0.88
	P1	0.9	0.3	
	Mean changes	0.2 ± 3	1.4 ± 1.3	0.65
HbA1c (%)	Baseline	11.3 ± 1.6	11.2 ± 1.3	0.84
	Week 10	11 ± 2	11.1 ± 1.8	0.82
	P1	0.3	0.3	
	Mean changes	− 0.3 ± 0.4	0.1 ± 0.5	0.65
HOMA-IR	Baseline	62 ± 63	53 ± 40	0.58
	Week 10	62.4 ± 42	65 ± 44	0.82
	P1	0.9	0.1	
	Mean changes	0.4 ± 21	12 ± 4	0.50
HOMA-B	Baseline	131 ± 119	128 ± 103	0.94
	Week 10	134 ± 98	140 ± 66	0.80
	P1	0.5	0.09	
	Mean changes	3 ± 21	12 ± 37	0.81
MDA (µmol/L)	Baseline	7.5 ± 1.6	7.5 ± 1.6	0.92
	Week 10	8 ± 1.7	8 ± 1.7	0.51
	P1	0.2	0.1	
	Mean changes	0.5 ± 0.1	0.5 ± 0.1	0.53
TAC (U/mL)	Baseline	0.81 ± 0.11	0.7 ± 0.1	0.58
	Week 10	0.8 ± 0.1	0.8 ± 0.1	0.70
	P1	0.2	0.4	
	Mean changes	− 0.01 ± 0.01	0.1 ± 0	0.07

P1: obtained from paired t-test P (p: 0.05)

P2: obtained from Independent sample t-test (p: 0.05)

## Discussion

In this randomized, double-blinded, placebo-controlled clinical trial, we indicated that curcumin supplementation has positive effect in reduction fasting blood glucose, body weight and hip circumference in patients with type 2 diabetes. Although there are some clinical trials that have described the effect of turmeric and its derivatives consumption on glycemic control, oxidative stress and anthropometric indices [3–8, 16, 17, 22–25], but most of these studies have been done on animal models and only a few of them have assessed the impact of curcumin on patients with diabetes and its complications. According to this study daily administration of 1500 mg curcumin could significantly reduce body weight and hip circumference in patients with type 2 diabetes. Also in one study it was showed, Supplementation with 500 mg curcumin for 4 weeks in obese children and adults showed a significant improve in serum adiponectin and lowered leptin levels that both of these factors play an important role in weight control [23]. Also in another clinical trial have been reported that consumption 1200 mg turmeric for a period of 8 weeks could reducing BMI in patients with type 2 diabetes [23]. There are different mechanisms for anti-obesity effects of curcumin, in its most important role declines the expression of genes involved in energy metabolism, lipid accumulation and reduction in intra cells fat [26]. Diet therapy with curcumin in rodent model causes body weight loss and raise lean body mass. In fact, curcumin inhibits the replication of proteins from the pathway of adipogenesis as well as curcumin leads to increase resting metabolic rate (RMR). This component of turmeric have direct effects on lipid metabolism such as reducing TG synthesis and increasing in beta oxidation of free fatty acids with raising metabolism rate and releases some kind of cytokines which are effective in weight loss. On the other hand curcumin inhibits genes expression of inflammatory cytokines like 1, 2, 6, 8, 12 Interleukins and Tumor necrosis factor alpha (TNF-α) by inactivate Nuclear Factor Kappa Beta (NF-ƙB) [27].

This trial demonstrate that daily supplementation with 1500 mg curcumin for 10 weeks could decrease fasting blood glucose in patients with type 2 diabetes, but no significant changes observed in serum insulin level, insulin resistance and HBA1c. According to the study conducted on 2013, receiving 300 mg curcuminoid daily for 3 months could reduce fasting blood glucose, HbA1c and HOMA-IR in participants with type 2 diabetes [28]. However daily supplementation with 2100 mg turmeric for 8 weeks had no significant effect on fasting blood glucose, serum insulin level and HbA1c [3]. There are various mechanisms for anti-diabetic effect of curcumin. One of the most fundamental of these mechanisms is that improvement in beta-cells function through its anti-inflammatory and anti-oxidant properties [7–9, 28]. In addition curcumin reduces fasting blood glucose by increasing Peroxisome proliferator-activated receptor (PPAR) activity, inhibits hyperglycemic, increases Glycolysis, inhibits liver gluconeogenesis, stimulations secretion insulin from pancreas, provokes glucose uptake by increasing gene expression of GLUT4, GLUT2 and GLUT3, suppresses liver production through improvement AMP kinase activation and inhibits glucose 6 phosphate kinase [16, 26, 28]. On the other hand curcumin increases the expression of adiponectin genes and thus leads to raising insulin sensitivity [29].

This investigation showed that receive 500 mg curcumin capsule 3 times a day for period of 10 weeks had no significant effect on reducing serum malondialdehyde level and increasing total antioxidant capacity. Oxidative stress reflects an imbalance between production of free radicals and body antioxidant defenses, for example Hyperglycemic could increase production of free radicals and oxidative stress [30]. Actually hyperglycemic through raising mitochondria free radicals production especially Reactive oxygen species (ROS) leads to oxidative stress in diabetes [31]. Curcumin as an antioxidant agent could enhance the expression of antioxidant enzymes and thus increases total antioxidant capacity. Mechanisms that are described for the antioxidant effects of curcumin include, Inhibition of glycosylation of proteins and lipid peroxidation, increased glutathione peroxidase activity, increasing the expression of antioxidant enzymes and enhancing the free radical clearing capacity [8].

In a study that investigated the effect of curcumin on MDA level, curcumin caused significant reduction on MDA level. In other study oral curcumin in diabetic rodent leaded to increase all antioxidant enzymes activity probably due to improve access to glutathione reductase, increased expression of Superoxidase dismutase, catalase and glutathione reductase [15].

The present study had some limitation. First, it seems that 10 weeks administration of curcumin there is not enough time to influence the factors involved in this trial also It was not possible to continue the longer intervention period due to the loss of samples and second, lake of access to nonocurcumin, another form of curcumin that can have significantly therapeutic effect and nanorange formulation. Also there are some reports linking curcumin with iron deficiency anemia. So patients with iron deficiency anemia and using curcumin supplements should be tested for possible causality.

## Conclusion

In conclusion our findings suggest that daily intake of 1500 mg curcumin powder reduces fasting blood glucose, weight and hip circumference in patient with type 2 diabetes, but had no effect on oxidative stress, serum insulin level, insulin resistance and HbA1c.

## Data Availability

Please contact authors for data request.

## Abbreviations

WHO: World Health Organization
BMI: body mass index
WC: waist circumference
FBS: fasting blood sugar
HbA1c: hemoglobin A1c
MDA: malondialdehyde
TAC: total antioxidant capacity
HOMA-IR: Homeostatic Model Assessment for Insulin Resistance
HOMA-B: pancreatic B-cell function
SBP: systolic blood pressure
DBP: diastolic blood pressure
RMR: resting metabolic rate
Tg: triglycerides
LDL-C: low density lipoprotein-cholesterol
TNF-α: tumor necrosis factor alpha
NF-ƙB: nuclear factor kappa beta
PPAR: peroxisome proliferator-activated receptor
ROS: reactive oxygen species

## References

[1] Organization WH (2016). World health statistics 2016: monitoring health for the SDGs sustainable development goals.

[2] Verma S, Hussain ME (2017). Obesity and diabetes: an update. Diabetes Metab Syndr.

[3] Adab Z, Eghtesadi S, Vafa M, Heydari I, Shojaei A, Haqqani H (2013). Effect of turmeric on body measurement indices, glycemic condition, and lipid profile in hyperlipidemic patients with type 2 diabetes. Iran J Nutr Sci Food Technol.

[4] Aggarwal BB, Kumar A, Bharti AC (2003). Anticancer potential of curcumin: preclinical and clinical studies. Anticancer Res.

[5] Patil T, Srinivasan M (1971). Hypocholesteremic effect of curcumin in induced hypercholesteremic rats. Indian J Exp Biol.

[6] Srivastava R, Puri V, Srimal R, Dhawan B (1986). Effect of curcumin on platelet aggregation and vascular prostacyclin synthesis. Arzneimittelforschung.

[7] Chuengsamarn S, Rattanamongkolgul S, Luechapudiporn R, Phisalaphong C, Jirawatnotai S (2012). Curcumin extract for prevention of type 2 diabetes. Diabetes Care.

[8] Jain SK, Rains J, Jones K (2006). Effect of curcumin on protein glycosylation, lipid peroxidation, and oxygen radical generation in human red blood cells exposed to high glucose levels. Free Radical Biol Med.

[9] Rivera-Mancía S, Lozada-García MC, Pedraza-Chaverri J (2015). Experimental evidence for curcumin and its analogs for management of diabetes mellitus and its associated complications. Eur J Pharmacol.

[10] Soetikno V, Suzuki K, Veeraveedu PT, Arumugam S, Lakshmanan AP, Sone H (2013). Molecular understanding of curcumin in diabetic nephropathy. Drug Discov Today.

[11] Son Y, Lee JH, Cheong Y-K, Chung H-T, Pae H-O (2013). Antidiabetic potential of the heme oxygenase-1 inducer curcumin analogues. BioMed Res Int.

[12] Bradford PG (2013). Curcumin and obesity. BioFactors.

[13] Aldebasi YH, Aly SM, Rahmani AH (2013). Therapeutic implications of curcumin in the prevention of diabetic retinopathy via modulation of anti-oxidant activity and genetic pathways. Int J Physiol Pathophysiol Pharmacol.

[14] Arun N, Nalini N (2002). Efficacy of turmeric on blood sugar and polyol pathway in diabetic albino rats. Plant Foods Hum Nutr.

[15] El-Bahr SM (2013). Curcumin regulates gene expression of insulin like growth factor, B-cell CLL/lymphoma 2 and antioxidant enzymes in streptozotocin induced diabetic rats. BMC Complement Altern Med.

[16] Ghorbani Z, Hekmatdoost A, Mirmiran P (2014). Anti-hyperglycemic and insulin sensitizer effects of turmeric and its principle constituent curcumin. Int J Endocrinol Metab.

[17] Gupta SK, Kumar B, Nag TC, Agrawal SS, Agrawal R, Agrawal P (2011). Curcumin prevents experimental diabetic retinopathy in rats through its hypoglycemic, antioxidant, and anti-inflammatory mechanisms. J Ocul Pharmacol Ther.

[18] Maradana MR, Thomas R, O’sullivan BJ (2013). Targeted delivery of curcumin for treating type 2 diabetes. Mol Nutr Food Res.

[19] Nishiyama T, Mae T, Kishida H, Tsukagawa M, Mimaki Y, Kuroda M (2005). Curcuminoids and sesquiterpenoids in turmeric (*Curcuma longa* L.) suppress an increase in blood glucose level in type 2 diabetic KK-Ay mice. J Agric Food Chem.

[20] Seo KI, Choi MS, Jung UJ, Kim HJ, Yeo J, Jeon SM (2008). Effect of curcumin supplementation on blood glucose, plasma insulin, and glucose homeostasis related enzyme activities in diabetic db/db mice. Mol Nutr Food Res.

[21] Kelishadi R, Rabiei K, Khosravi A, Famouri F, Sadeghi M, Rouhafza H (2001). Assessment of physical activity of adolescents in Isfahan. J Shahrekord Univ Med Sci..

[22] Ismail NA, Abd-El-Dayem SM, Salama E, Ragab S, Abd-El-Baky AN, Ezzat WM (2016). Impact of curcumin intake on gluco-insulin homeostasis, leptin and adiponectin in obese subjects. Res J Pharm Biol Chem Sci.

[23] Maithilikarpagaselvi N, Sridhar MG, Swaminathan RP, Zachariah B (2016). Curcumin prevents inflammatory response, oxidative stress and insulin resistance in high fructose fed male Wistar rats: potential role of serine kinases. Chem Biol Interact.

[24] Muniyappa R, Lee S, Chen H, Quon MJ (2008). Current approaches for assessing insulin sensitivity and resistance in vivo: advantages, limitations, and appropriate usage. Am J Physiol Endocrinol Metab.

[25] Song Z, Wang H, Zhu L, Han M, Gao Y, Du Y (2015). Curcumin improves high glucose-induced INS-1 cell insulin resistance via activation of insulin signaling. Food Funct.

[26] Alappat L, Awad AB (2010). Curcumin and obesity: evidence and mechanisms. Nutr Rev.

[27] Shehzad A, Ha T, Subhan F, Lee YS (2011). New mechanisms and the anti-inflammatory role of curcumin in obesity and obesity-related metabolic diseases. Eur J Nutr.

[28] Na LX, Li Y, Pan HZ, Zhou XL, Sun DJ, Meng M (2013). Curcuminoids exert glucose-lowering effect in type 2 diabetes by decreasing serum free fatty acids: a double-blind, placebo-controlled trial. Mol Nutr Food Res.

[29] Weisberg SP, Leibel R, Tortoriello DV (2008). Dietary curcumin significantly improves obesity-associated inflammation and diabetes in mouse models of diabesity. Endocrinology.

[30] Venkateswaran S, Pari L, Saravanan G (2002). Effect of Phaseolus vulgaris on circulatory antioxidants and lipids in rats with streptozotocin-induced diabetes. J Med Food.

[31] Meng B, Li J, Cao H (2013). Antioxidant and antiinflammatory activities of curcumin on diabetes mellitus and its complications. Curr Pharm Des.

